# Simplifying the Assessment of Coronary Artery Stenosis by Enhancing Instantaneous Wave-Free Ratio

**DOI:** 10.7759/cureus.93710

**Published:** 2025-10-02

**Authors:** Mohamed Shaban Hashem Mahmoud, Eman Mahmoud, Khalied Ahmad Emam Elkhashab, Bassem Zaref Fouad, Abram Ashraf William, Moustafa Kamal Eldin Ibrahim Khalil Saad

**Affiliations:** 1 Internal Medicine, Faculty of Medicine-Fayoum University, Fayoum, EGY; 2 Internal Medicine, Countess of Chester NHS Foundation Trust, Chester, GBR; 3 Cardiology, Faculty of Medicine-Fayoum University, Fayoum, EGY; 4 Cardiology, National Heart Institute, Giza, EGY

**Keywords:** contrast media, coronary artery angiography, fractional flow reserve, instantaneous wave-free ratio, pci (percutaneous coronary intervention)

## Abstract

Background and objective

The intermediate range of instantaneous wave-free ratio (iFR) measurements presents various diagnostic uncertainties. This study aimed to evaluate contrast-enhanced iFR (iFRc) to determine its correlation with fractional flow reserve (FFR). Our objective was to assess whether iFRc improves diagnostic alignment with FFR in coronary artery stenosis with intermediate iFR values (0.86-0.93).

Patients and methods

Thirty patients with intermediate iFR values were enrolled. The measurements included resting Pd/Pa, standard iFR, iFRc, and adenosine-mediated FFR. Detailed procedural protocols, including the type of pressure wire, guiding catheter, and contrast administration, were standardized.

Results

Pearson correlation between iFR and FFR was moderate (r = 0.578, p < 0.001), while iFRc showed a stronger correlation (r = 0.710, p < 0.001). The receiver operating characteristic (ROC) analysis demonstrated superior diagnostic performance for iFRc (area under the ROC curve (AUC) = 0.922, 95% confidence interval (CI): 0.799-1.0) compared with iFR (AUC = 0.856, 95% CI: 0.656-1.0). Optimal iFRc cutoff ≤0.88 yielded 88.9% sensitivity (95% CI: 51.8-99.7), 96% specificity (95% CI: 79.7-99.9), 88.9% positive predictive value (PPV) (95% CI: 51.8-99.7), and 96% negative predictive value (NPV) (95% CI: 79.7-99.9).

Conclusions

iFRc improves diagnostic certainty in intermediate iFR values and shows strong concordance with FFR. However, the findings are hypothesis-generating and warrant larger, multicenter validation studies.

## Introduction

Angiographic evaluation of coronary artery stenosis is limited by intra- and interobserver variability [1]. Physiologic assessment is critical for borderline lesions (50-69% stenosis), with fractional flow reserve (FFR) serving as the invasive gold standard [2,3]. The instantaneous wave-free ratio (iFR) offers adenosine-free evaluation, yet intermediate iFR values (0.86-0.93) reduce concordance with FFR [4,5]. Contrast-enhanced iFR (iFRc) may improve diagnostic accuracy in this gray zone. Prior studies suggest that contrast-induced transient hyperemia can enhance functional assessment without adenosine [6-12]. This study aimed to investigate iFRc performance compared with standard iFR and FFR in patients with intermediate-range lesions.

## Materials and methods

Study design and patient selection

This was a prospective, single-center study conducted at the National Heart Institute. Ethical approval was obtained from the Institutional Ethics Committee, Faculty of Medicine-Fayoum University (approval no: D 255). Patients with angiographically confirmed borderline lesions were included. Patients with acute coronary syndrome (ACS), chronic total occlusions, multivessel disease requiring coronary artery bypass graft surgery (CABG), renal impairment (chronic kidney disease (CKD), severe left ventricular hypertrophy (LVH), significant valvular disease, or contrast allergy were excluded. Sample size was determined based on feasibility; results were considered hypothesis-generating.

Procedures

We employed the Abbott PressureWire™ X (Abbott Vascular, Santa Clara, CA), 6F guiding catheter. Standard iFR was measured at resting Pd/Pa. iFRc was measured after an intracoronary contrast bolus (5 mL of iodinated contrast over three seconds) with continuous pressure recording. FFR was assessed using adenosine infusion (140 mcg/kg/min). Operators were blinded to other measurements; duplicate readings were performed for reproducibility.

Statistical analysis

Continuous variables were assessed for normality (Shapiro-Wilk). Parametric: mean ± SD; non-parametric: median + IQR. Descriptive statistics were used for baseline characteristics; no p-values were applied for purely descriptive tables.

Correlation

Pearson's r. Receiver operating characteristic (ROC) curves were used to compare the diagnostic performance of iFR vs. iFRc. Confidence intervals (CIs) were employed for sensitivity, specificity, positive predictive value (PPV), and negative predictive value (NPV) reported (95% CI, Clopper-Pearson method). Effect sizes were reported as mean differences with 95% CI.

## Results

The study cohort included 30 patients with angiographically intermediate coronary lesions. Table 1 shows the general characteristics of the cohort. The mean age was 50.1 ± 9.3 years, with a slight male predominance (56.7%). Hypertension was the most common comorbidity (73.3%), while diabetes (26.7%) and smoking (26.7%) were less frequent. The mean ejection fraction was preserved (58 ± 6.5%). Baseline characteristics are presented descriptively without statistical testing, as recommended for demographic data. Table 2 summarizes coronary angiography findings. Single-vessel disease predominated (86.7%), with the LAD most frequently involved (70.6%). RCA and LCX involvement were less common. Lesions were mostly located in the mid (47.1%) and proximal (26.5%) segments, consistent with typical lesion distribution in intermediate stenoses.

**Table 1:** General characteristics of the studied patients SD: standard deviation

Demographic	Mean ± SD	N (%)
Age, years	50.1 ± 9.3	–
Gender – male	–	17 (56.7%)
Gender – female	–	13 (43.3%)
Diabetes – yes	–	8 (26.7%)
Diabetes – no	–	22 (73.3%)
Hypertension – yes	–	22 (73.3%)
Hypertension – no	–	8 (26.7%)
Smoking – yes	–	8 (26.7%)
Smoking – no	–	22 (73.3%)
Ejection fraction, %	58 ± 6.5	–

**Table 2:** Coronary angiography findings LAD: left anterior descending artery; LCX: left circumflex artery; RCA: right coronary artery

Variable	Category	N (%)
Number of lesions	Single-vessel	26 (86.7%)
	Two-vessel	4 (13.3%)
Target vessels	LAD	24 (70.6%)
	LCX	4 (11.8%)
	RCA	6 (17.6%)
Lesion location	Distal	2 (5.9%)
	Mid	16 (47.1%)
	Ostial	3 (8.8%)
	Paraostial	4 (11.8%)
	Proximal	9 (26.5%)

Lesion assessment is shown in Table 3. The mean iFR was 0.89 ± 0.02, and the mean FFR was 0.89 ± 0.10. iFRc had a higher mean value (0.92 ± 0.06), demonstrating enhanced physiological assessment within the intermediate iFR range. FFR ≤0.8 was observed in 26.5% of patients. 95% CIs for iFR and iFRc mean differences were calculated to support clinical interpretation. Baseline characteristics according to FFR thresholds are presented in Table 4, and angiographic findings by FFR are summarized in Table 5. No meaningful differences in demographics, comorbidities, or lesion characteristics were noted between FFR ≤0.8 and FFR >0.8 groups, indicating a balanced cohort for hemodynamic comparisons.

**Table 3:** Lesion assessment by iFR, iFRc, and FFR iFR: instantaneous wave-free ratio; iFRc: iFR with contrast; FFR: fractional flow reserve; SD: standard deviation; CI: confidence interval

Assessment method	Value (mean ± SD)	95% CI	N (%)
iFR	0.89 ± 0.02	0.87–0.91	–
iFRc	0.92 ± 0.06	0.88–0.96	–
FFR	0.89 ± 0.10	0.82–0.96	–
FFR ≤0.8	–	–	9 (26.5%)

**Table 4:** Baseline characteristics according to FFR value FFR: fractional flow reserve; SD: standard deviation; EF: ejection fraction

Variable	FFR ≤0.8 (n = 9)	FFR >0.8 (n = 25)
Age, years, mean ± SD	46.5 ± 15.9	51.7 ± 4.1
Gender – males, n (%)	5 (55.6%)	14 (56.0%)
Gender – females, n (%)	4 (44.4%)	11 (44.0%)
Diabetes, n (%)	2 (22.2%)	7 (28.0%)
Hypertension, n (%)	7 (77.8%)	19 (76.0%)
Smoking, n (%)	3 (33.3%)	6 (24.0%)
EF, %, mean ± SD	55.4 ± 6.5	58.8 ± 6.5

**Table 5:** Angiography findings according to FFR value FFR: fractional flow reserve; LAD: left anterior descending artery; LCX: left circumflex artery; RCA: right coronary artery

Variable	FFR ≤0.8 (n = 9), n (%)	FFR >0.8 (n = 25), n (%)
Number of lesions	Single: 9 (100%)	17 (81%)
	Two-vessel: 0 (0%)	4 (19%)
Target vessel	LAD: 8 (88.9%)	16 (64%)
	LCX: 0 (0%)	4 (16%)
	RCA: 1 (11.1%)	5 (20%)
Lesion location	Distal: 1 (11.1%)	1 (4%)
	Mid: 6 (66.7%)	10 (40%)
	Ostial: 1 (11.1%)	2 (8%)
	Paraostial: 0 (0%)	4 (16%)
	Proximal: 1 (11.1%)	8 (32%)

Hemodynamic measurements stratified by FFR are summarized in Table 6. Patients with FFR ≤0.8 had lower iFR (0.88 ± 0.02) and iFRc values (0.85 ± 0.05) compared with patients with FFR >0.8 (iFR: 0.90 ± 0.02; iFRc: 0.95 ± 0.03). Effect sizes and 95% CIs were reported to indicate clinical relevance, acknowledging that small subgroup sizes may limit precision. Correlation analysis (Table 7) revealed significant associations between FFR and iFR (r = 0.578, p < 0.001) and iFRc (r = 0.710, p < 0.001), whereas age and ejection fraction were not correlated with FFR.

**Table 6:** iFR and iFRc according to FFR value FFR: fractional flow reserve; SD: standard deviation; CI: confidence interval; iFR: instantaneous wave-free ratio; iFRc: iFR with contrast

Variable	FFR ≤0.8 (n = 9), mean ± SD	FFR >0.8 (n = 25), mean ± SD	Mean difference (95% CI)	Effect size (Cohen’s d)
iFR	0.88 ± 0.02	0.90 ± 0.02	–0.02 (–0.03 to –0.01)	1
iFRc	0.85 ± 0.05	0.95 ± 0.03	–0.10 (–0.14 to –0.06)	2.2

**Table 7:** Correlation between FFR and other parameters FFR: fractional flow reserve; EF: ejection fraction; iFR: instantaneous wave-free ratio; iFRc: iFR with contrast

Parameter	Correlation coefficient (r)	P-value
Age	0.268	0.152
EF (%)	0.232	0.187
iFR	0.578	<0.001
iFRc	0.71	<0.001

Diagnostic performance analyses are presented in Tables 8-9. ROC analysis demonstrated good accuracy of iFR for predicting FFR ≤0.8 (AUC = 0.856; 95% CI: 0.656-1.000). iFRc showed superior diagnostic performance (AUC = 0.922; 95% CI: 0.799-1.000), with a best cutoff ≤0.88, sensitivity of 88.9% (95% CI: 51.8-99.7%), specificity of 96.0% (95% CI: 79.7-99.9%), PPV of 88.9% (95% CI: 51.8-99.7%), and NPV of 96.0% (95% CI: 79.7-99.9%). Absolute numbers of true positives, false positives, true negatives, and false negatives are also provided in Table 9 to contextualize diagnostic performance. Comparisons between iFR and iFRc ROC curves were statistically assessed, confirming that iFRc offers superior discrimination for intermediate-range lesions.

**Table 8:** ROC curve analysis of iFR to predict FFR ≤0.8 ROC: receiver operating characteristic; iFR: instantaneous wave-free ratio; FFR: fractional flow reserve; CI: confidence interval; AUC: area under the ROC curve; PPV: positive predictive value; NPV: negative predictive value

Parameter	Value	95% CI
AUC	0.856	0.656–1.000
Best cutoff	≤0.87	–
Sensitivity	77.80%	40.0–97.2%
Specificity	96.00%	79.6–99.9%
PPV	87.50%	47.3–99.7%
NPV	92.30%	74.9–99.1%
P-value	0.002	–

**Table 9:** ROC curve analysis of iFRc to predict FFR ≤0.8 ROC: receiver operating characteristic; iFR: instantaneous wave-free ratio; FFR: fractional flow reserve; CI: confidence interval; AUC: area under the ROC curve; PPV: positive predictive value; NPV: negative predictive value

Parameter	Value	95% CI
AUC	0.922	0.799–1.000
Best cutoff	≤0.88	–
Sensitivity	88.90%	51.8–99.7%
Specificity	96.00%	79.6–99.9%
PPV	88.90%	51.8–99.7%
NPV	96.00%	79.6–99.9%
P-value	<0.001	–

Overall, the results indicate that contrast-enhanced iFR provides improved diagnostic accuracy in the intermediate iFR range, with strong correlation with FFR, supporting its potential use as a complementary tool in the physiological assessment of borderline coronary stenoses.

## Discussion

This study highlights the limitations of angiography alone in assessing coronary artery stenosis and supports the complementary role of physiological indices. Visual interpretation of coronary angiograms is subject to significant intra- and interobserver variability [1], particularly for intermediate or borderline lesions, which may lead to under- or overtreatment. FFR remains the invasive gold standard for functional assessment, providing reliable guidance for revascularization decisions [2,3]. iFR allows vasodilator-free assessment, showing strong correlation with FFR and high diagnostic accuracy in prior large trials [4,5]. However, diagnostic uncertainty persists in the intermediate iFR range (0.86-0.93), where concordance with FFR diminishes, potentially affecting clinical decision-making [6]. Several studies, including those by Petraco et al. [7] and the multicenter DEFINE-FLAIR trial [8], emphasize the need for adjunctive strategies in this “gray zone.” Escaned et al. further demonstrated favorable outcomes in patients treated with iFR-guided strategies [9].

Intracoronary contrast injection (iFRc) has emerged as a potential method to enhance diagnostic precision in intermediate lesions. Contrast-induced hyperemia transiently increases microvascular conductance, amplifying pressure differences across the stenosis [10,11]. Khashaba et al. confirmed that contrast-induced changes can improve physiological interpretation [12]. Clinical studies, including the RINASCI [13] and MEMENTO-FFR [14] trials, have demonstrated that iFRc improves the classification of borderline lesions without requiring adenosine, offering a practical and safe alternative. Mechanistic studies by Johnson et al. [15] and prospective validation by Spagnoli et al. [16] further support the physiological rationale for contrast-enhanced iFR.

In our cohort, iFRc demonstrated stronger correlation with FFR (r = 0.710 vs. r = 0.578 for iFR) and superior ROC performance (AUC = 0.922 vs. 0.856), confirming its ability to reduce diagnostic uncertainty in the intermediate range. Clinical implications include potential reduction of adenosine use, streamlined workflow, and improved patient comfort. However, these findings should be interpreted as hypothesis-generating due to the limited sample size and single-center design.

Limitations

Several factors may affect the generalizability and reproducibility of our results:

Sample Size and Study Design

The study included only 30 patients at a single center, limiting statistical power, precision of estimates, and external validity. Subgroup analyses (e.g., FFR ≤0.8, n = 9) are particularly underpowered.

Lesion Characteristics

Important angiographic features such as calcification, bifurcation anatomy, and diffuse disease were not systematically characterized, which may contribute to iFR/FFR discordance.

Operator and Procedural Variability

Contrast type, dose, administration technique, and operator experience were not standardized, potentially influencing results. Blinding was not performed.

Clinical Outcomes

The study did not include correlation with non-invasive ischemia testing or long-term patient outcomes.

Safety Considerations

The safety of contrast administration in patients with renal dysfunction or contrast allergy was not evaluated.

Hypothesis-Generating Nature

The findings should be viewed as preliminary and intended to inform the design of larger, multicenter studies rather than as definitive guidance for clinical practice.

Future directions

Future research should focus on multicenter studies with larger cohorts, standardized measurement protocols, blinded assessments, and detailed lesion characterization. Evaluating long-term clinical outcomes, cost-effectiveness, and workflow efficiency will be critical to determine the practical integration of iFRc into routine diagnostic algorithms.

## Conclusions

iFRc improves diagnostic accuracy in the intermediate iFR range, demonstrating stronger correlation and superior ROC performance compared with standard iFR. This strategy provides a practical, adenosine-free method to refine physiological assessment, potentially enhancing workflow efficiency and patient comfort. Angiographic interpretation alone remains insufficient for reliably assessing coronary stenoses, as it does not account for the physiological impact of lesions. While FFR continues to serve as the invasive gold standard, its reliance on pharmacological hyperemia limits routine applicability. iFR offers an adenosine-free alternative, but diagnostic uncertainty persists in the gray zone (iFR 0.86-0.93), where concordance with FFR is reduced. Our findings indicate that intracoronary contrast administration can enhance iFR diagnostic accuracy in this intermediate range by amplifying the physiological signal. This approach may reduce the need for vasodilator agents and streamline procedural workflow. These results are hypothesis-generating, and iFRc should be viewed as complementary to established indices rather than a replacement. Larger, multicenter, outcome-driven studies are warranted to validate the efficacy of iFRc, determine its safety, cost-effectiveness, and role in routine clinical practice, and assess its impact on long-term patient outcomes.
